# Feedback control of collective dynamics in an oscillator population with time-dependent connectivity

**DOI:** 10.3389/fnetp.2024.1358146

**Published:** 2024-02-02

**Authors:** Michael Rosenblum

**Affiliations:** Institute of Physics and Astronomy, University of Potsdam, Potsdam, Germany

**Keywords:** network physiology, oscillations, connectivity, non-autonomous dynamics, synchrony control

## Abstract

We present a numerical study of pulsatile feedback-based control of synchrony level in a highly-interconnected oscillatory network. We focus on a nontrivial case when the system is close to the synchronization transition point and exhibits collective rhythm with strong amplitude modulation. We pay special attention to technical but essential steps like causal real-time extraction of the signal of interest from a noisy measurement and estimation of instantaneous phase and amplitude. The feedback loop’s parameters are tuned automatically to suppress synchrony. Though the study is motivated by neuroscience, the results are relevant to controlling oscillatory activity in ensembles of various natures and, thus, to the rapidly developing field of network physiology.

## 1 Introduction

The merger of control science and nonlinear dynamics ideas led to essential achievements in controlling chaos, coherence of noisy dynamics, and noise-induced motion; see (Schöll and Schuster, 2008) for a review. Another field of application is controlling the level of synchrony. As is known, synchronization is a general and frequently encountered phenomenon that may be beneficial or harmful (Winfree, 1967; Kuramoto, 1984; Pikovsky et al., 2001; Strogatz, 2003; Osipov et al., 2007). So, synchronizing low-power generators helps to sum their outputs and thus create a high-power source; see, e.g., (Tiberkevich et al., 2009). Synchronization is vital for the stable operation of power grids (Dörfler and Bullo, 2012) and cardiac pacemakers (Jalife, 1984). Examples of an opposite value stem from neuroscience, where pathologically increased synchrony is believed to be a cause of, e.g., Parkinson’s disease (Tass, 1999; Tass, 2000) and pedestrian bridge dynamics (Dallard et al., 2001; Eckhardt et al., 2007). This paper addresses the case of a negative impact. It discusses suppressing undesired synchrony in a highly interconnected population of active, self-sustained oscillators - a problem of general interest for network physiology (Ivanov, 2021).

Two approaches to this problem are known from the literature: the open-loop (Tass, 1999; 2001; 2002) and closed-loop control (Rosenblum and Pikovsky, 2004a; b; Popovych et al., 2005; Lin et al., 2013; Wilson and Moehlis, 2015; Holt et al., 2016; Zhou et al., 2017). The most known and tested open loop scheme is the coordinated reset (Tass, 2003; Popovych and Tass, 2012; Adamchic et al., 2014; Manos et al., 2021; Munjal et al., 2021; Khaledi-Nasab et al., 2022); it requires stimulation via multiple electrodes. The feedback schemes exploit one measuring and one stimulating electrode. Furthermore, the feedback techniques can be categorized as continuous-time and pulsatile methods. Here, we concentrate on the latter case, suitable for neuroscience applications such as deep brain stimulation (DBS) (Benabid et al., 1991; 2009; Kühn and Volkmann, 2017), where only stimulation by pulses is possible.

The main features of our modeling study are as follows. 1) We suggest a network model with time-dependent global connectivity to imitate brain activity data with strong amplitude modulation. 2) We consider that one cannot measure the rhythm of interest directly but shall extract it from its mixture with other rhythms and noise. 3) We implement a realistic scheme for real-time estimating the signal’s phase and amplitude. 4) We suggest an adaptive control approach to tune the stimulation on the fly. Our main result is that we automatically find two vulnerable phases per oscillation period and achieve suppression of the strongly modulated activity by stimulation at these phases only.

In the rest of this section, we briefly review the main results in the feedback-based manipulation of the ensemble synchrony and specify our problem in more detail.

### 1.1 Feedback control of collective synchrony: state of the art

All known closed-loop techniques assume one can monitor the activity in question and stimulate at least a large part of the oscillator population. The population is supposed to be highly interconnected, so the mean-filed approximation is justified. If continuous-time stimulation is possible, one achieves the desired goal by feeding back a delayed or phase-shifted measurement (Rosenblum and Pikovsky, 2004b; Tukhlina et al., 2007). The explanation is simple: elements of the population maintain synchrony due to forcing by the mean field. If stimulation compensates for the mean field, the oscillators naturally desynchronize due to frequency inhomogeneity and individual noisy perturbations. It means the stimulation shall be approximately in antiphase to the measurement and have roughly the same amplitude. Thus, the problem reduces to determining the proper phase shift and amplification; this can be done by trial and error or by an adaptive control scheme (Montaseri et al., 2013). The paramount property of this technique is the stimulation tends to zero, or practically to the level of noise, as soon as the control goal is achieved and the undesired rhythm declines. Therefore, the scheme is denoted as the vanishing stimulation. In a practical situation, one measures a mixture of the activity to be suppressed with other rhythms and noise, so filtration shall be a part of the feedback scheme.

Another way to describe the mechanism of the continuous feedback stimulation is to assume that the collective mode appears via the Hopf bifurcation and write the corresponding normal form equation. Linear delayed or non-delayed feedback (Rosenblum and Pikovsky, 2004b; Tukhlina et al., 2007) changes the linear term and thus stabilizes the otherwise unstable origin. On the level of individual oscillators, this corresponds to the asynchronous state. The normal form representation clarifies the effect of the nonlinear feedback (Popovych et al., 2005): it changes the nonlinear term in the normal form equation, thus reducing the collective mode’s amplitude without causing the bifurcation.

In many applications, especially in neuroscience, continuous-time stimulation is not feasible and has to rely on a pulsatile feedback scheme. The most straightforward solution is to employ the continuous feedback signal to modulate a high-frequency pulse train (Popovych et al., 2017). Another approach is to modulate the pulses’ amplitude following the measurement but stimulate only at the vulnerable phases (Rosenblum, 2020); in this way, one can essentially reduce the intervention in the system. In this paper, we follow this path and simulate the desynchronizing feedback accounting for such practical issues as extracting the signal of interest from its mixture with noise and real-time estimation of the signal’s phase and amplitude. We suggest a network model with time-dependent connectivity to mimic the time course of data registered from Parkinsonian subjects and demonstrate a successful synchrony control of such a network by appropriately timed charge-balanced pulses. Next, we propose and discuss an algorithm for automatically tuning feedback parameters.

Before modeling the pulsatile feedback-based desynchronization, we discuss why certain oscillation phases are vulnerable. Consider a limit-cycle motion subject to an external stimulus that acts along some unknown direction. This direction depends on the system’s equations and how the stimulus enters these equations. Since we do not know the system’s equations, we cannot predict how the given stimulus influences the oscillation amplitude. However, we can say that the stimulus pushes the system off the limit cycle. If we apply the stimulus at such a phase that it pushes the system towards the unstable steady state inside the cycle, this action is optimal for desynchronization. Indeed, the ensemble’s synchronous state corresponds to the collective activity’s limit-cycle oscillation, while the asynchronous state corresponds to a fixed-point solution. Thus, there exists an optimal, vulnerable phase *θ*_0_. Obviously, stimulation at *θ*_0_ + *π* with a stimulus of opposite polarity is also optimal. Unfortunately, we cannot guess *θ*_0_. We can only apply stimuli at different phases and look at the response.

## 2 Modeling the closed-loop desynchronization

### 2.1 Simulating the amplitude-modulated activity

A commonly used model of collective dynamics in a highly interconnected oscillatory network is a mean-field coupled population. In computational neuroscience, the models frequently incorporate two groups of units, modeling excitatory and inhibitory neurons; in this case, the coupling organization may be more complex. The common feature of the models from this class is that they produce a nearly periodic collective mode. The deviation from the periodicity is due to the finite-size effect and, sometimes, weak collective chaos that is more pronounced if individual units are chaotic. However, the envelope of the rhythm to be suppressed does not vanish, and currently available techniques successfully treat this case. We concentrate here on the case when the activity of our interest is waxing and waning, as frequently observed in neuroscience measurements [for an example, see the time plots of band-passed beta-band activity from a patient with Parkinson’s disease in (Rosenblum et al., 2021)].

We use a phenomenological model to simulate the waxing and waning patterns when the activity bursts are interrupted by quiescent epochs. We take *N* globally coupled heterogeneous^1^ Bonhoeffer–van der Pol oscillators:

$$\left\{\begin{array}{cc} {\dot{x}}_{k}\; & =x_{k}-x_{k}^{3}/3-y_{k}+I_{k}+\varepsilon \left(t\right)X+\cos\psi \cdot P\left(t\right)\;,\\ {\dot{y}}_{k}\; & =0.1\left(x_{k}-0.8y_{k}+0.7\right)+\sin\psi \cdot P\left(t\right)\;, \end{array}\right.$$ (1)

where *k* = 1, …, *N* is the oscillator index, and the coupling is organized via the mean field *X* = *N* ^−1^ *∑*_*k*_ *x*_*k*_. Function *P*(*t*) describes external stimulation, and the parameter *ψ* quantifies how the stimuli affect the system and determines the phase beneficial for stimulation. For the following, we fix *ψ* = *π*/4. In the simulation of the feedback loop, this parameter is considered unknown, and a search algorithm is used to find an appropriate stimulation phase.

**FIGURE 1:**
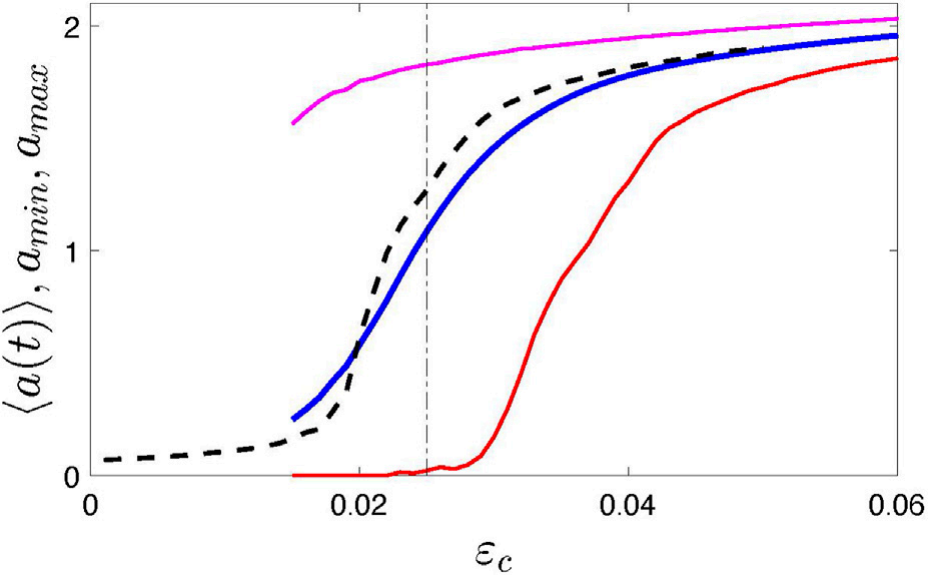
Synchronization transition in system (1) illustrated by the dependence of the time-averaged amplitude ⟨*a*(*t*)⟩ on the coupling strength. Black dashed line presents the standard case when the coupling is time-independent, i.e., Δ*ɛ* = 0. Blue, red, and magenta solid lines show the averaged, minimal, and maximal amplitude, respectively. Vertical dotted-dashed line indicates the value *ɛ*_*c*_ = 0.025 used in the following; this value corresponds to the strongly modulated mean field with vanishing envelope, see time plots in Figure 4.

A new feature of the model is the time-dependent coupling strength *ɛ*(*t*). We assume that it fluctuates around some central value *ɛ*_*c*_. For simulations, we use a simple algorithm; namely, we let *ɛ*(*t*) be a piece-wise constant function of time, *ɛ*(*t*) = *ɛ*_*n*_ = const for *t*_*n*_ ≤ *t* < *t*_*n*+1_ = *t*_*n*_ + *τ*_*n*_, where *ɛ*_*n*_ and *τ*_*n*_ are random numbers, chosen from a uniform distribution between *ɛ*_*c*_ ∓Δ*ɛ* and *τ*_min_, *τ*_max_, respectively. Figure 1 illustrates the synchronization transition in the ensemble of *N* = 1,000 elements for Δ*ɛ* = 0.015, *τ*_min_ = 200 and *τ*_max_ = 500. Here, for the order parameter, we take the oscillation amplitude^2^, averaged over a long time interval, ⟨*a*(*t*)⟩. We see, that for 0.015 ≲*ɛ*_*c*_ ≲ 0.03 we obtain strongly modulated mean field *X*. The time plots are given in the Results section below. For comparison, we also show the synchronization transition for the usual globally-coupled model with *ɛ* = const (formally, it corresponds to Δ*ɛ* = 0). For simulations, we exploited the fourth-order Runge-Kutta scheme with the time step 0.1.

The suggested model is certainly purely phenomenological. However, it is natural to assume that, e.g., neuronal networks in the brain are not frozen but permanently altered, e.g., due to plasticity (Manos et al., 2021; Asl et al., 2022; Khaledi-Nasab et al., 2022). Real-world networks are nonautonomous, adaptive (Gross and Blasius, 2008; Berner et al., 2023), and subject to internal fluctuations and external perturbations. Though the account of all details is impossible, a phenomenological model can help to describe the dynamics close to the synchronization transition point.

### 2.2 Real-time preprocessing

#### 2.2.1 Measurement and filtering

Measuremental noise is inevitable. This noise and some irrelevant rhythms are often intense and contaminate the useful signal, so filtration becomes necessary before phase and amplitude estimation. For real-time applications, the filter can use only the current and previous measurement points, i.e., it must be causal. Thus, we implement a finite impulse response bandpass filter of length 2*M* + 1 points. The filter length *M* determines its quality: a longer digital filter provides better signal attenuation for the frequencies outside the desired band. Finite impulse response filters have linear phase response, which means they do not distort the signal but delay it by *M* points. Thus, the filter’s output is delayed by *Mδ*, where *δ* is the sampling rate. Hence, we can obtain only delayed values of the instantaneous phase and amplitude. If the delay is small, we can estimate the actual phase as *θ*(*t*) ≈ *θ*(*t* − *Mδ*) + *ωMδ*, where *ω* is the average oscillation frequency. However, the larger the delay, the worse the estimation because the system is noisy and exhibits phase diffusion. Thus, the filter shall not be too long. On the other hand, short filters are not efficient. Hence, there is an optimal filter semilength *M*.

#### 2.2.2 Causal phase and amplitude estimation

Here, we implement an algorithm exploiting a non-resonant linear oscillator described in (Rosenblum et al., 2021). The idea is as follows. Suppose, for a moment, our signal is harmonic, and we use it to drive a linear damped oscillator; we use this oscillator as a virtual “measuring device.” The phase and amplitude of the oscillator are related to those of the driving force by well-known resonance curve formulas. Since we know the state of the linear oscillator, we invert the resonance relations to obtain the phase and amplitude of the force, i.e., the phase and amplitude of the analyzed signal. The crucial step is to choose the proper parameters for the “device.” By choosing the linear oscillator’s frequency much larger than the signal’s frequency and selecting appropriate damping, we can ensure that the amplitude and frequency response is approximately flat in a large interval of input frequencies. We need two oscillators to achieve this: a strongly-damped one yields a nearly constant amplitude response for frequencies much smaller than the resonant one, while a weakly-damped one provides a nearly constant phase response. The algorithm also works for the force with slowly varying amplitude and frequency. Practically, we substitute each linear oscillator with the corresponding differential equation and solve it numerically for the discretely-spaced input. Reference (Rosenblum et al., 2021) provides an efficient numerical scheme. The approach has demonstrated its efficiency in tests with bandpassed beta activity of Parkinsonian subjects (Busch et al., 2022).

### 2.3 Stimulation

**FIGURE 2:**
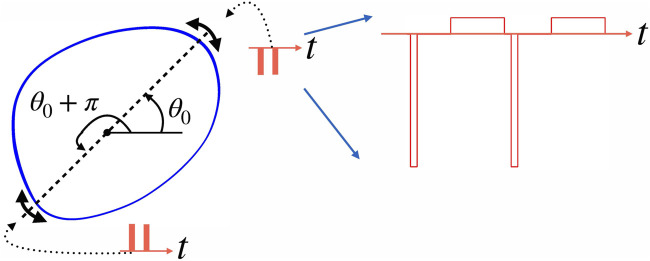
This schematic figure illustrates the algorithm of stimulation at vulnerable phases. Let such phase be *θ*_0_. It means that a negative pulse applied at this phase pushes the point on the limit cycle (blue closed curve) towards the unstable steady state inside the cycle (filled circle), and so does a positive stimulus applied at *θ*_0_ + *π*. Practically, we check whether the circular distance between the current phase *θ*(*t*) and optimal phase *θ*_0_ is smaller than the tolerance parameter *α*. So, the system is stimulated if the phase is in the vicinity of *θ*_0_ or *θ*_0_ + *π*, as indicated by double-headed arrows. Generally, several stimuli (shown by red bars) fulfill this condition; for this illustration, we assume that this number is two. The right part of the figure shows the actual stimulus shape used in simulations. Here, two negative stimuli are shown. Each stimulus consists of a narrow but high rectangular pulse followed after some gap by a compensating pulse that is low but wide.

We model stimulation by short stimuli. Thus, *P*(*t*) = *∑*_*n*_ *p*(*t* − *t*_*n*_), where *t*_*n*_ are the instants of stimulus’s application and *p*(*t*) describes the pulse shape. The stimuli have a finite length *T*_*s*_, i.e., *p*(*t*) = 0 for *t*∉[0, *T*_*s*_]. In many neuroscience applications, an additional requirement applies: the stimuli must be charge-balanced to avoid charge accumulation in the live tissue. Thus, we use bipolar stimuli consisting of two rectangular pulses of opposite polarity and equal area to fulfill the condition $\int_{0}^{T_{s}}p\left(t\right)dt=0$ . Notice that, generally, there is a gap between two monopolar pulses. We apply them when the collective oscillation phase is close to the optimal one (for the moment, we assume that this optimal phase *θ*_0_ is known). Namely, we check whether the circular distance between *θ* and *θ*_0_ is smaller than tolerance *α*. We also introduce the minimal interval Δ between the pulses. In dependence on *α*, Δ, there can be one or several stimuli when phase *θ*(*t*) crosses the *α*-vicinity of *θ*_0_. Next time, the stimulation is turned on when *θ* ≈ *θ*_0_ + *π*, within the tolerance *α*. We illustrate the algorithm in Figure 2.

Next, we discuss the intensity of the stimulation. We implement a feedback algorithm with the feedback factor *ɛ*_*fb*_ < 0. It means the signal’s instantaneous amplitude *a*(*t*) determines the stimulation amplitude *A*_*n*_, i.e., the height of the narrow rectangular pulse of the *n*th stimulus: *A*_*n*_ = *A*(*t*_*n*_) = max(*ɛ*_*fb*_ *a*(*t*), − *A*_0_) for stimulation around *θ*_0_ and *A*_*n*_ = *A*(*t*_*n*_) = −max(*ɛ*_*fb*_ *a*(*t*), − *A*_0_) for stimulation around *θ*_0_ + *π*.^3^ The parameter *A*_0_ determines the maximal allowed stimulation amplitude.

Until now, we assumed that the optimal (vulnerable) phase *θ*_0_ and the feedback factor *ɛ*_*fb*_ are known. In an actual experiment, we must determine these parameters, i.e., we need an adaptive control scheme. A natural approach is to try stimulation with different *θ*_0_, *ɛ*_*fb*_ and look at whether the signal’s amplitude is enhanced or suppressed. The known algorithms (Montaseri et al., 2013; Rosenblum, 2020) work well for signals without strong amplitude modulation, both for time-continuous and pulsatile stimulation. However, our tests find that these adaptation schemes become unstable for strong modulation, i.e., in the case treated in this paper.

We solve the problem by exploiting a trial-and-error algorithm. It means we try to stimulate at various phases and look when the stimulation is most efficient. However, this is not that trivial for the case of a burst-like, amplitude-modulated signal because we cannot tell the amplitude reduction due to stimulation from that due to internal dynamics. Indeed, the data’s essential feature is that the amplitude decreases practically to zero from time to time without any stimulation. On the other hand, even if we stimulate at the proper phase, the amplitude can grow due to increased coupling within the oscillator population. We approach the problem as follows.

In the first step, we do not stimulate but calculate the autonomous system’s average amplitude *a*_*aut*_. Next, in the learning phase, we start with some initial values for *ɛ*_*fb*_ and then gradually change *θ*_0_ from zero to 2*πN*_*cycl*_. Namely, we stimulate as described for *m* = 5 oscillation periods and then check whether *a*_*curr*_ < *a*_min_. Here, *a*_*curr*_ is the current amplitude value, averaged over *m* periods, and *a*_min_ is the minimal amplitude value (Initially, we set *a*_min_ = 0.3*a*_*aut*_.) If *a*_*curr*_ < *a*_min_, we set *a*_min_ = *a*_*curr*_ and choose the current value for the optimal phase as *θ*_*opt*_ = *θ*_0_. Then, we stimulate the next *m* cycles and check the condition *a*_*curr*_ < *a*_min_ again. If *a*_*curr*_ > *a*_min_ but decreases, we make no changes and continue stimulation; otherwise we adjust the parameters as

$$\theta_{0}\mapsto \theta_{0}+\text{max}\left(\Delta \theta ,\Delta \theta \cdot a_{\mathrm{curr}}/a_{\mathrm{aut}}\right)\;,$$

where Δ*θ* is the maximal allowed step, e.g., Δ*θ* = 2*π*/25, and

$$\varepsilon_{fb}\mapsto \varepsilon_{fb}-f\left(\varepsilon_{fb}\right)\;,$$ (2)

where *f* is a decreasing function of |*ɛ*_*fb*_|. Using such a function, we avoid feedback that is too strong. For real-time applications, it is beneficial to choose a computationally efficient function; we choose $f\left(x\right)∼{\left(1+cx^{2}\right)}^{-1}$ , where *c* is a parameter. This described step implements the trial-and-error search: if amplitude reduction accompanies the stimulation, we keep the stimulation parameters unchanged. Otherwise, we adjust them. We proceed with the learning epoch unless *θ*_0_ reaches 2*πN*_*cycl*_ and then take the current values *θ*_*opt*_ and *ɛ*_*fb*_ for the following. (If we never achieved *a*_min_ < 0.3*a*_*aut*_ during the learning epoch, we force the adaptation algorithm to perform one more learning cycle.) Thus, the length of the learning epoch is determined by the number *N*_*cycl*_ of complete cycles of variation of *θ*_0_; Figure 7 in the next Section illustrates the dependence of the results on *N*_*cycl*_. After the learning epoch, we keep optimal phase constant, continue tracing the signal’s amplitude, and adapt the feedback factor according to Eq. 2 if *a*_*curr*_ > 2*a*_min_.

## 3 Results

In this section, we show the results of feedback-based suppression of the noise-contaminated mean field *X*(*t*) generated by the globally coupled population described by Eq. 1. For the observable used by the control scheme, we take *X*_*N*_ = *X* + *σξ*, where *ξ* is white Gaussian noise with zero average and standard deviation one. We fix *σ* = 3, i.e., the noise is strong.

**FIGURE 3:**
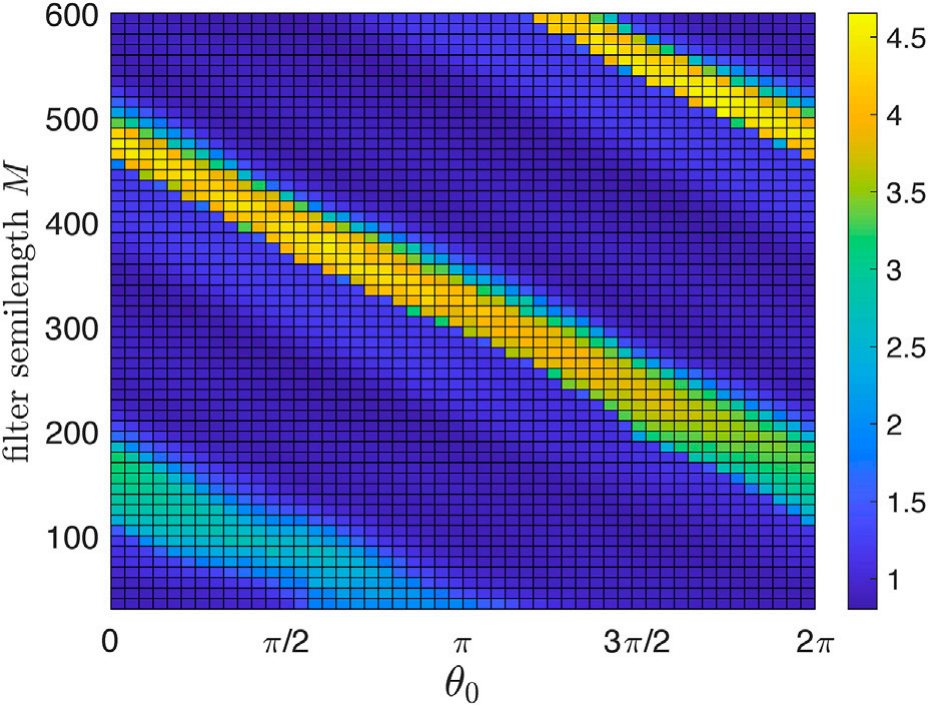
Suppression factor (color-coded) as a function of the stimulation phase *θ*_0_ and causal filter semilength *M*. Short filters are not efficient and, therefore, yield poor suppression. Large *M* ensures much better filtration but increases the delay introduced by the filter and thus spoils the overall performance of the feedback desynchronization scheme. The optimal *M* is about 350. For larger *M*, the maximal suppression factor remains nearly the same, but the algorithm becomes more sensitive to the choice of *θ*_0_.

As an auxiliary step, we demonstrate in Figure 3 the effect of the digital filter.^4^ Above, we argued that there is an optimal filter semilength *M*. Here, we show the suppression degree in dependence on *M*. To concentrate on this issue, we consider the deterministic case of Eq. 1 with *ɛ* = const = 0.03. We scan *M* and *θ*_0_ and show by color coding the suppression factor *S* = *S*(*θ*_0_, *M*). We define the suppression factor as the ratio of the autonomous system’s standard deviation over the stimulated system’s standard deviation (the latter is computed after a sufficiently long transient). As expected, short filters are inefficient, while for long filters, the stimulation setup becomes more sensitive to the choice of *θ*_0_. For the following, we choose *M* = 350; for this filter, good suppression is achieved for 0.64*π* ≲*θ*_0_ ≲ 0.84*π*.

**FIGURE 4:**
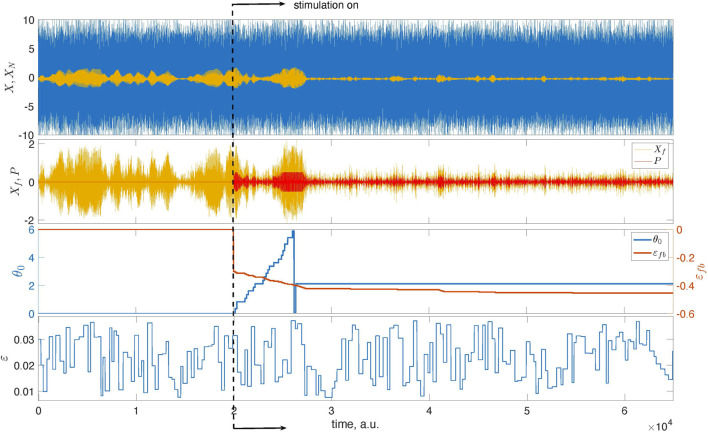
Illustration of the feedback-based pulsatile desynchronization. The top panel presents the mean field *X* of system (1) and its mixture with noise, *X*_*N*_. The latter is used as the input to the feedback loop. The second panel from the top presents *X*_*f*_, that is, the filtered series *X*_*N*_, and the stimulation that is turned on at *t* = 2 ⋅ 10^4^. At this instant, the learning epoch begins. During this epoch, the stimulation phase *θ*_0_ grows from zero to 2*π*. After that, the stimulation continues with the optimal value of *θ*_0_. The bottom panel presents the time-dependent coupling.

Figure 4 presents the main results. Now, we exploit the model with the time-dependent coupling with parameters *ɛ*_*c*_ = 0.025 and Δ*ɛ* = 0.015.^5^ We run the unstimulated system for *t* < 2 ⋅ 10^4^, then turn on the stimulation and successfully suppress the collective oscillation. We take *N*_*cycl*_ = 1 for the adaptation epoch. The estimated optimal phase *θ*_*opt*_ = 0.67*π* corresponds to the domain of efficient suppression for the chosen filter.

**FIGURE 5:**
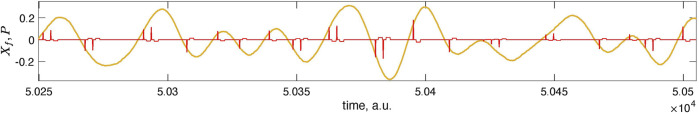
Here, we magnify the short epoch of the data from Figure 4 (second panel from the top). In this magnification, one can see individual stimuli. Negative polarity pulses appear near the optimal phase *θ*_*opt*_, while positive pulses appear near *θ*_*opt*_ + *π*. There are one or two pulses in the neighborhood of the vulnerable phase for the chosen parameters.

**FIGURE 6:**
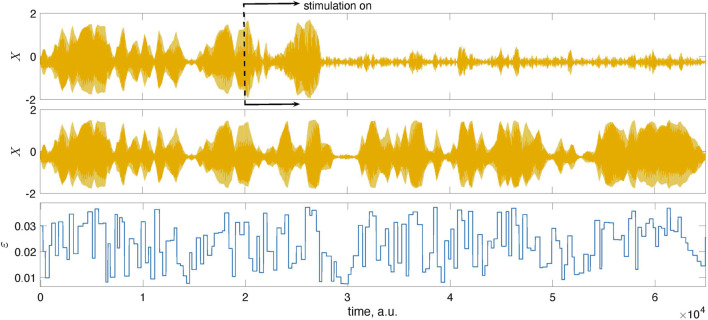
The mean fields *X* of the population (1) shown here were obtained from two different runs: in the top panel, the stimulation was turned on at the instant indicated by the dashed line, while in the middle panel, we show the results for the autonomous, unstimulated system. The system was started with the same initial conditions in both runs and simulated using the same time-dependent coupling. Naturally, for *t* < 2 ⋅ 10^4^, the signals coincide. For larger times, they differ due to stimulation.

Figure 5 and Figure 6 provide further illustrations for this test. In Figure 5, we show a short epoch of the data from Figure 4 for the suppressed state magnified so that one sees individual stimuli. Figure 6 proves that the decrease of the amplitude is indeed due to stimulation and not internal dynamics. To demonstrate this, we run the system twice, with the same initial conditions and the same realization of the random time-dependent coupling *ɛ*(*t*). However, we switch on the stimulation in the first case while the system remains autonomous in the second.

**FIGURE 7:**
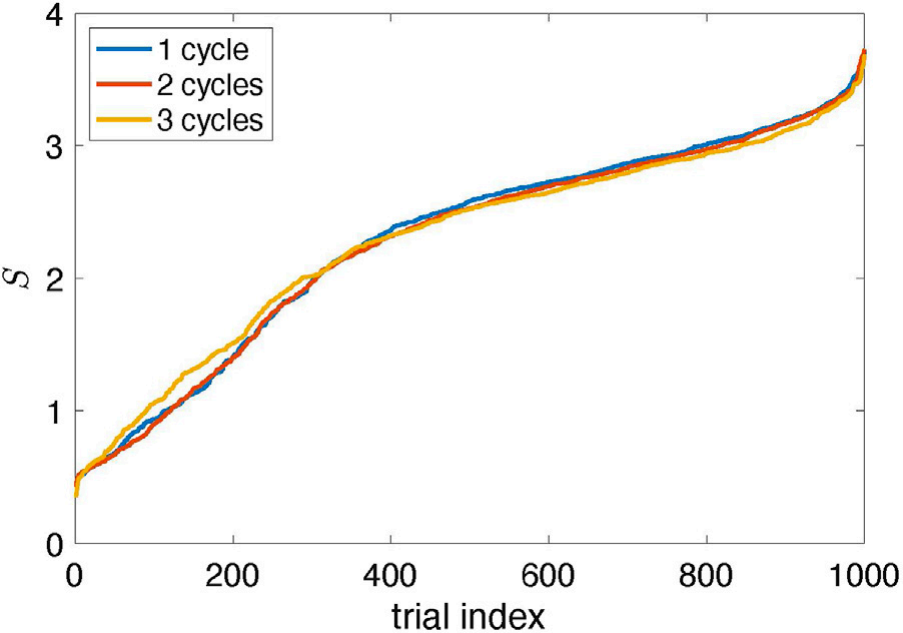
Suppression factor obtained in 1,000 trials with different realizations of the random time-dependent coupling *ɛ*(*t*) and for *N*_*cycl*_ = 1, 2, 3; it means that in the learning epoch, the stimulation phase *θ*_0_ changed from zero to 2*πN*_*cycl*_ (The data are sorted in ascending order.) Approximately 90% of trials yield suppression of the collective mode (*S* > 1); about 70% provide significant suppression (*S* > 2).

The natural question is whether the suggested algorithm always finds the proper stimulation parameters. The answer is no. The results in Figure 4 are typical, but sometimes the algorithm fails. Since it traces the amplitude variation in response to stimulation, the errors are inevitable because the amplitude can occasionally reduce due to internal dynamics, not stimulation. To estimate the probability of a failure, we performed 1,000 runs with different random realizations of the time-dependent coupling and computed the suppression factor *S*. We repeated this test with two and three learning cycles, letting *θ*_0_ in the learning phase grow to 4*π* and 6*π*, respectively. The results shown in Figure 7 demonstrate that the stimulation algorithm fails in ≈10% of runs (*S* < 1, the amplitude increases) and provides essential suppression (*S* > 2) in ≈70% of trials.We also see that adaptation with several learning cycles is not helpful: the performance in the case of three cycles is only slightly better than for one cycle.

## 4 Discussion

We presented a detailed simulation of the pulsatile feedback-based control of collective synchrony, concentrating on the case when the observed dynamics exhibit strong amplitude modulation; this case is relevant, e.g., for neuroscience. To simulate such modulated activity, we introduced a model of an oscillatory network with global randomly fluctuating coupling. We assumed that the input to the feedback loop is a mixture of the ensemble mean field and broad-band noise of high intensity. We demonstrated an efficient network desynchronization by incorporating a bandpass filter into the feedback loop and using an adaptation algorithm to tune the stimulation setup. We emphasize that we stimulate at vulnerable phases only and reduce the stimulation amplitude as soon as the goal is achieved, thus minimizing the intervention into the system. This property may be helpful for applications in life sciences and network physiology (Ivanov, 2021).

The results show that contamination of the rhythm in question by other spectrally separated rhythms and noise is not an obstacle. The feedback loop compensates for the delay introduced by a causal bandpass digital filter. Substituting the simple filter used in our simulations with a more advanced one [see, e.g. (Smetanin et al., 2020)] may improve the overall performance.

The algorithm finding the optimal stimulation phase and feedback factor can possibly be improved. One option to enhance its performance is to restart the learning epoch after some time if the suppression factor after the first search for optimal parameters is too small. An alternative approach is to substitute the adaptation algorithm by inference of the amplitude response of the system (Duchet et al., 2020; Cestnik et al., 2022) using specially designed test stimulation; the inferred information can then be used for suppressing stimulation.

Our simulations use bipolar charge-balanced stimuli consisting of two rectangular pulses with a gap in between. As known (Popovych O. V. et al., 2017; Popovych and Tass, 2019), the gap influences the stimulation efficiency, and optimizing the stimulus’s shape remains challenging (Wilson and Moehlis, 2014; Mau and Rosenblum, 2022). We notice that the desynchronization task simplifies without this application-specific charge balance requirement, i.e., when monopolar stimuli are allowed.

Finally, we stress that we paid particular attention to the computational efficiency of all building blocks of the control loop. Neither the phase and amplitude estimation nor optimal parameters search require functions computation but only arithmetical operations. Thus, the feedback scheme can be used in real-time applications and easily implemented in specialized hardware. Therefore, the presented results can be helpful in ongoing experimental research on phase-specific deep brain stimulation (Rosin et al., 2011; Cagnan et al., 2013; 2017; Little et al., 2013; Holt et al., 2019; McNamara et al., 2020). As a possible direction for future studies, we mention a further development of a network neuronal model exhibiting amplitude-modulated, burst-like behavior that will account for different types of plasticity, external inputs, internal fluctuations, and other realistic properties of the pathological pacemaker.

## Data Availability

The raw data supporting the conclusion of this article will be made available by the authors, without undue reservation.

## Footnotes

1 Parameters *I*_*k*_ determine oscillator frequencies; they are taken from a Gaussian distribution with the mean 0.6 and standard deviation 0.1. These parameters ensure that all units are in the oscillatory state and have different frequencies.

2 For the amplitude calculation we exploit the MATLAB^®^ envelope function; this function uses the spline interpolation between the local maxima to provide a smooth envelope of a time series.

3 We remind that we compute the instantaneous amplitude for the filtered and, hence, time-delayed signal.

4 We used the MATLAB^®^ fir1 function.

5 Parameters of the stimuli are: width of the first pulse is 0.2, gap is 1, and width of the compensating pulse is 1.6; the minimal distance between the pulses is Δ = 0.2. Further parameters are: *α* = 0.1*π*, *A*_0_ = 0.5.
